# Risky Sexual Practices, Sexually Transmitted Infections, Motivations, and Mental Health among Heterosexual Women and Men Who Practice Sexualized Drug Use in Spain

**DOI:** 10.3390/ijerph19116387

**Published:** 2022-05-24

**Authors:** Daniel Íncera-Fernández, Francisco J. Román, Manuel Gámez-Guadix

**Affiliations:** Department of Personality, Assessment and Psychological Treatment, Autonomous University of Madrid, 28049 Madrid, Spain; daniel.incera@estudiante.uam.es (D.Í.-F.); f.javier.roman@uam.es (F.J.R.)

**Keywords:** chemsex, sexualized drug use, heterosexual, mental health, sexual behavior, public health

## Abstract

Sexualized drug use (SDU) has been poorly studied among heterosexuals. The purpose of the present study was to analyze the prevalence of and gender differences in types of substances, risky sexual practices, sexually transmitted infections (STIs), motivations, and psychological adjustment among heterosexual women and men who engage in SDU. The study sample consisted of 1181 heterosexuals (795 women) between 18 and 78 years old (mean age = 24.4, *SD* = 7.4). Approximately 12% of the participants had engaged in SDU. No differences were found in the prevalence of SDU between men and women. Alcohol, cannabis, and 3,4-methylenedioxy-methamphetamine (MDMA) were the substances most frequently used for sexual purposes. Men were significantly more likely to use MDMA, ecstasy, cocaine, and erectile dysfunction (ED) drugs, and they tended to have more sexual partners than women. Likewise, SDU was related to have more sexual partners, penetrative sex without a condom, practice a fetish, be diagnosed with syphilis, chlamydia, and others STIs, and present more depression symptoms (but not with more anxiety). In conclusion, SDU was associated with poorer physical and mental health. It is, therefore, necessary to design programs aimed at reducing the incidence of the consequences of SDU on the physical and mental health of both men and women. Moreover, programs that seek to understand why these individuals engage in SDU should be undertaken.

## 1. Introduction

Sexualized drug use (SDU), which includes the phenomenon of “chemsex” as a particular subset of substances, refers to the intentional use of drugs before or during sexual intercourse [1,2]. Men who have sex with men (MSM) have been found to practice SDU to increase euphoria, sexual arousal, and intense sexual experiences as well as to manage inhibition and increase confidence to perform certain sexual practices [3,4]. SDU can lead to risky sexual behaviors [5] and an increased risk of STIs [6,7], including the human immunodeficiency virus (HIV; [8]). Additionally, SDU could be associated with mental health risks (e.g., depression; [9,10]). Most studies on SDU have been conducted among MSM [11]. Empirical studies of male and female heterosexuals are very scarce [12], and whether drugs were used before or during sexual intercourse has not been analyzed [13]. Thus, additional investigation is required on the practice of SDU among heterosexuals, including women and men, to understand the characteristics of this phenomenon and to design information and intervention strategies relating to the possible risks to sexual and mental health from a broader, holistic approach. The purpose of this study, therefore, was to extend the previous empirical evidence by analyzing the prevalence and gender differences in the type of substances, risky sexual practices and STIs, motivations, and mental health outcomes among heterosexual people who practiced SDU. Next, we review the existing empirical evidence on SDU.

### 1.1. Sexualized Drug Use (SDU)

Substances commonly used to enhance sexual experiences in MSM include gamma-hydroxybutyrate/gamma-butyrolactone (GHB/GBL; [14]), 4-methylmethcathinone (mephedrone; [15]), n-methyl-1-phenylpropan-2-amine (methamphetamine; [16]), ketamine [17], cocaine [18], MDMA (“ecstasy”; [19]), alkyl and butyl nitrites (poppers; [8]), erectile dysfunction drugs [17], cannabis [20], and alcohol [21]. Estimates of the prevalence of SDU among MSM vary widely between different studies, ranging from 6% to 90% [22,23]. Likewise, there is no agreement in the research about which substances are consumed most frequently. While the systematic review by Maxwell et al. (2019) points out that MDMA was the most used substance, other studies find that poppers [7], alcohol [24], GHB/GBL [14], methamphetamine [25], or mephedrone [26] were the drugs most used among MSM. These differences could be due to the different substances considered in “SDU” between studies or to the different characteristics of the samples. Although research to date has focused more often on the sex-related drug use of lesbian, gay, bisexual, and transgender (LGBT) people compared to use by heterosexual communities, some studies indicate that the use of certain substances could also be frequent in both men and women, regardless of their sexual orientation [18,27]. For example, Lawn et al. (2019) found that alcohol, cannabis, and MDMA were most frequently used in sexual relationships by heterosexual women and men. Little is known, however, about gender differences in SDU among heterosexuals. In addition, limited data are available on the specific drugs used during SDU by heterosexual women and men. This study will help us gain a more accurate vision of the differential consumption patterns among genders and allow us to tailor intervention programs and prevention campaigns by adopting a gender perspective. 

### 1.2. SDU, Risky Sexual Behaviors, and STIs

Several studies have shown that SDU decreases risk perception [3,28] and has been associated with riskier sexual practices [29,30,31]. Among the most common risk practices among sexualized drug users are the reduced use of condoms [32,33], multiple casual sexual partners [34,35], and unconventional sexual practices (i.e., bondage and discipline, dominance and submission, and sadism and masochism (BDSM); fisting, barebacking, or serosorting; [36,37]), which could also facilitate the transmission of HIV [38]. For example, different studies focused on drug use and STI risk have associated the risk of acquiring HIV in both men and women with a higher frequency of risky sexual relations [39,40]. According to Bellis et al. (2008) [41], risky sexual practices while under the effect of psychoactive substances are related to the alteration in decision making produced by drug use, which, in turn, increases the probability of unprotected sex. Although strong associations between SDU and risky sexual behaviors have been reported, few data are currently available for heterosexual women and men.

### 1.3. Motivations for Using Substances for Sexual Purposes

Several motivations exist for SDU among MSM [12]. These motivations include enhancing sexual desire, increasing sexual arousal and pleasure, facilitating long sex sessions (i.e., sessions lasting several hours or days), engaging in unconventional sexual practices, promoting disinhibition, or fostering social ties by sharing intimate spaces among MSM [42,43]. While some people identify SDU as an escape route from difficult and painful situations or as a way to manage problems in general [44,45], other MSM point out that SDU is a strategy used to regulate negative emotions due to internalized homophobia or feelings generated by the stigma associated with HIV infection [45,46]. While the motivations for SDU have been studied primarily among MSM, some studies have looked at the motivations for practicing SDU in heterosexual women and men. Among the scarce literature, Cheng et al. (2010) [47] found different motivations for consuming methamphetamine among their study participants: experiencing new sensations (42%), being alert or more energetic (41%), improving sex and meeting new sexual partners (31%), and losing weight (20%). Possible gender differences in motivations for participating in SDU, however, have not been examined to date.

### 1.4. Mental Health and Drug Use for Sexual Purposes

The use of psychoactive substances has frequently been related to a high incidence, prevalence, and severity of mental health outcomes (e.g., Borodovsky and Budney, 2018 [48]). Although psychological symptoms of people who use drugs for sexual purposes has increased in recent years [49,50], studies of the variables associated with mental health are still scarce [51]. Tomkins et al. (2019) [52] carried out a systematic review, finding that SDU is associated with symptoms such as depression, anxiety, and addiction to recreational drugs in MSM. Similarly, a recent study by Miltz et al. (2021) found that drug use is also associated with symptoms of anxiety and depression in heterosexual men and women. Still, other research has found no relationship between SDU and psychological adjustment [20,53]. 

Recently, some substances commonly used to enhance sexual experiences (e.g., MDMA and quetiapine) have been the subjects of research, especially in studies regarding their therapeutic utility for treating anxiety and depression [54,55]. Although these substances are potentially toxic, the beneficial effects would be mediated by the dose and the route of administration [51]. The extent to which psychological adjustment is associated with SDU among heterosexuals, as well as possible differences in psychological adjustment between genders, is unknown.

### 1.5. The Present Study

Studies of SDU among MSM have increased substantially in recent years (e.g., Tomkins et al., 2019). However, the empirical evidence of SDU among heterosexual persons is still considerably scant. The first objective of this study, therefore, was to analyze gender differences in the prevalence of SDU by evaluating the different types of substances used during sexual relations between heterosexuals. SDU studies have focused on MSM, so the risks of STIs related to the use of drugs during sexual relationships between heterosexuals has been scarcely examined and not sufficiently addressed in STI prevention strategies among heterosexuals. Therefore, our second objective was to analyze SDU in heterosexual women and men and its association with risky sexual behaviors and self-reported STIs. Similarly, little is known about possible motivations for heterosexual drug use and whether these motives differ between women and men. Therefore, the third objective of this study was to analyze motivations for using drugs in sexual contexts among heterosexual women and men. Finally, little is known about mental health outcomes associated with SDU in heterosexual users. Therefore, our last objective was to analyze the association between SDU in heterosexual women and men and their symptoms of depression and anxiety.

## 2. Materials and Methods

### 2.1. Study Design and Participants

The initial sample comprised 1520 participants. Considering the aims of this study, 339 questionnaires were discarded (22.30%). Those participants who did not self-identify as heterosexual (*n* = 305), did not reside in Spain (*n* = 23), or had more than 20% missing values were excluded (*n* = 11). Data were collected anonymously using an online, self-administered, cross-sectional survey from February to May 2021. The survey site received a total of 2293 visits. The response rate was 51.50% (N = 1181). Heterosexual women and men aged 18 and over were invited to participate through messages on social networks (i.e., Facebook, Instagram, and Twitter) and personal messages (i.e., WhatsApp). The inclusion criteria to participate in the present study were as follows: (a) be at least 18 years old; (b) be a man, woman, heterosexual, or nonbinary person; and (c) have resided in Spain for the last 24 months. Those who decided to access the questionnaire were directed to an initial screen that informed them about the objectives and content of the study. Participants were informed that they could choose not to answer the questions and that study participation could be discontinued at any time and for any reason without consequences. No incentives were offered to participants in exchange for their participation. All study data were collected anonymously. The survey took approximately 10–20 min to complete. Participants were given the contact e-mail of the researchers in case they had any questions about the survey content or the study. This study followed the ethical standards and norms of the Declaration of Helsinki.

### 2.2. Measures

#### 2.2.1. Sociodemographic Characteristics

Participants were asked to provide a set of sociodemographic characteristics as part of the study data. Those characteristics included their gender, age, country of birth, educational level, employment situation, monthly income, age of first sexual encounter, whether they had a stable partner, and whether they had sex outside the couple relationship.

#### 2.2.2. Substance Use for Sexual Purposes

To explore drug use, we asked participants if, during the last 24 months, they had used drugs or substances before or during a sexual encounter to facilitate, intensify, or prolong sexual activity. SDU was defined as the intentional use of drugs (i.e., alcohol, cannabis, cocaine, poppers, ecstasy, ED medication, MDMA, GHB/GBL, methamphetamine, mephedrone, heroine, benzodiazepine, and other substances) for sex. The names of the drugs were listed in the questionnaire. A broad definition of substances used for sexual purposes was used since, according to the existing literature, substances such as alcohol, cannabis, and cocaine have also been associated with the practice of SDU [56,57,58]. An open category of “other substances” was added, in which participants could specify which drug they used if they felt it was not included in the proposed options. In addition, participants were asked to provide the mean amount of money that was invested monthly for the acquisition of substances that were associated with improving sexual experiences.

#### 2.2.3. Sexual Practices and STIs

Participants were asked the number of their casual sexual partners and the frequency of condom use in penetrative sex with casual partners (they answered “not applicable” when they had not had penetrative sex with casual partners). Participants were also asked to report the use of fetishes or unconventional sexual practices, such as BDSM (i.e., erotic practice based on the immobilization of a person’s body), fisting (i.e., sexual conduct that consists of the introduction of the hand or part of the arm into the anus/rectum/vagina), barebacking (i.e., condomless sex), serosorting (i.e., sexual partners chosen based on their HIV status), or other practices. These questions were chosen based on previous quantitative studies that examined risky sexual practices associated with SDU [32,35,59]. In addition, participants were asked to indicate whether they had been diagnosed with any STIs in the last 24 months.

#### 2.2.4. Motivations

Participants were asked to select their motivations for practicing SDU. The options for motivations included: (a) “to evade and/or have fun,” (b) “to disinhibit myself and feel less ashamed,” (c) “to have sex, perform certain sexual practices or feel that I perform better sexually,” (d) “to feel closer to others and be intimate,” (e) “so I don’t feel lonely and isolated,” and (f) “for the feeling that you have to use well the spare time having fun.” These responses were developed based on qualitative research previously conducted on the motivations of MSM for participating in SDU [43,44,53].

#### 2.2.5. Mental Health

The mental health measure included depression and anxiety subscales of the Brief Symptom Inventory (BSI; [60]). Each subscale contains six items (i.e., symptoms). The participants indicated how often they had experienced each symptom of depression (e.g., feeling lonely, feeling blue, feeling no interest in things, feeling hopeless about the future, feeling worthless, thoughts of ending your life) and anxiety (nervousness or shakiness inside, suddenly scared for no reason, feeling fearful, feeling tense or keyed up, spells of terror or panic, feeling so restless that you cannot sit still) during the last 2 weeks on a scale from 1 (not at all) to 5 (a lot). The BSI has shown good psychometric properties in the Spanish population [61]. In this sample, internal consistency was adequate for both the depression subscale (Cronbach’s α = 0.82) and the anxiety subscale (Cronbach’s α = 0.83).

### 2.3. Data Analyses

We analyzed the data using IBM SPSS™ Statistics 26. First, we performed a descriptive analysis of the main characteristics of the participants, differentiating between those who had participated in SDU and those who had not in the last 24 months as a function of gender. To assess the differences between sociodemographic variables, risky sexual practices, and self-reported STI diagnoses between drug users for sexual purposes and nonusers, we used Pearson’s Chi-square test. The Chi-square test was also used to study differences between heterosexual women and men in substance use, risky sexual behaviors, self-reported STI diagnoses, and motivations for practicing SDU. The result of the Chi-squared does not provide detailed information regarding the differences among various combinations of groups. Therefore, we performed Bonferroni post hoc analyses to clarify the differences between specific pairs of experimental groups. In cases where there was a cell number smaller than five participants, the *p*-values were corrected using Fisher’s exact test. Finally, the Student’s *t*-test for independent samples and Cohen’s *d* were used to examine the differences in self-reported mental health between SDU and nondrug users and as a measure of effect size, respectively.

## 3. Results

### 3.1. Sociodemographic Characteristics

The final sample consisted of 1181 participants between 18 and 78 years old (*M* = 24.47, *SD* = 7.47). Among them, 795 (67.3%) were women, 382 (32.3%) were men, and a minority identified themselves as nonbinary (*n* = 4, <1%). Age did not differ significantly between women (*M* = 24.48, *SD* = 7.54) and men (*M* = 24.46, *SD* = 7.35; *t* = 0.062, *p* = 0.697). 

### 3.2. Prevalence and Sociodemographic Variables Associated with SDU

Of the total sample, 12.4% of the participants practiced SDU: 11.7% of the women (n = 93) and 13.9% of the men (*n* = 53) consumed some substance for sexual purposes (χ^2^ = 1.125, *p* = 0.289). Table 1 shows the prevalence of the participants who were and were not involved in SDU as well as their sociodemographic characteristics. Statistically significant differences were found between SDU and non-SDU in the variables of age, country of birth, and sex outside the couple. Specifically, post-hoc comparisons (*p* < 0.05, Bonferroni correction) showed that in the 26–35 age group, the practice of SDU is more common than in people ≥46 years (17.6% vs. 2.2%). Regarding place of birth, people born outside Spain practiced more SDU than those born in Spain (29.5% vs. 11.18%). Finally, people who had sex outside of a couple tended to practice SDU (25.6%) more than people without a partner (12.4%) or those who did not have sex outside of a couple (11.0%). The rest of the sociodemographic variables (i.e., educational level, income, and stable partner) did not differ significantly with respect to the consumption of SDU.

### 3.3. Relationship between SDU and Gender

Alcohol was the most frequently reported substance used to practice SDU (10.5%) in the previous 24 months, followed by cannabis (7.1%), MDMA (1.8%), ecstasy (1.4%), and cocaine (1.1%). The rest of the drugs had a prevalence of use < 1.0%. Men who engaged in SDU used MDMA, ecstasy, and cocaine more frequently than did women (3.1% and 1.1%, χ^2^ = 5.944, *p* < 0.05; 2.6% and 0.9%, χ^2^ = 5.471, *p* < 0.05; 2.1% and 0.6%, χ^2^ = 5.072, *p* < 0.05; 0.5% and 0.0%; see Table 2). Regarding money spent monthly for the acquisition of drugs for practicing SDU, 9.1% of the participants reported spending less than EUR 50 per month (see Table 2). Post-hoc comparisons showed significant gender differences for the following categories: “Between €100 and €200” (men > women) and “Nothing, they usually invite me” (women > men).

### 3.4. Relationship of SDU with Risky Sexual Behaviors and STIs

The results regarding risky sexual behaviors and STIs are presented in Table 3. The proportion of the participants with risky sexual behaviors in the last 24 months was higher among those who engaged in SDU compared to those who did not (χ^2^ = 42.667, *p* < 0.001). The post-hoc comparison showed that the participants who engaged in SDU were more likely to have had more than 20 casual sexual partners than those who did not engage in SDU (2.7% and 0.6%, respectively). This pattern of outcomes is also found when comparing the participants who had 10–20 or 5–10 casual sex partners (see Table 3). The frequency with which condoms were used appears to be associated with SDU (χ^2^ = 22.463, *p* < 0.001). These differences are found in the following categories: (1) “Sometimes,” in which the participants who performed SDU have a higher percentage than those who did not engage in SDU (22.6% vs. 13.2%), and “Not applicable, I have not had penetrative sex with casual partners,” in which the participants who performed SDU have a lower percentage than those who did not engage in SDU (8.9% vs. 19.4%).

Slightly more than 14% of all the participants reported having performed some unconventional or fetish sexual practice. Of these, the participants who had practiced SDU were significantly likelier to practice a fetish (27.0%) than those who did not use drugs for sexual purposes (12.4%; χ^2^ = 21.119, *p* < 0.001). Compared to those who had not done so, those who had done SDU in the previous 24 months performed BDSM more frequently (7.1% and 14.3%, respectively; χ^2^ = 9.173, *p* < 0.01), and barebacking (4.6% and 10.9%, respectively; χ^2^ = 9.784, *p* < 0.01). In addition, we analyzed gender differences for variables relating to unsafe sexual practices. The results show that men who practiced SDU were more likely to have more than 20 sexual partners (5.8%) than women (1,1%; χ^2^ = 15.479, *p* < 0.01). More women than men who practiced SDU (37.7% and 21.7%, respectively) “always” used a condom in their penetrative sex, although the differences were marginally significant (χ^2^ = 10.800, *p* = 0.055). The use of fetishes in sexual relationships did not differ significantly with respect to the gender of the participants in the SDU.

When the participants were asked if they had been diagnosed with any STI in the last 24 months, 5.3% of the sample reported that they had been diagnosed with at least one STI. The participants who had practiced SDU were more commonly diagnosed with some STI (11.6%) than those who had not used (4.4%; χ^2^ = 12.905, *p* < 0.001). Compared with the participants who did not use the drugs for sexual purposes, those who did use them more commonly indicated having been diagnosed with syphilis or chlamydia in the previous 24 months (see Table 3). The occurrence of other STIs did not differ significantly with respect to the use or absence of sexualized substances. STIs also did not differ as a function of gender among the participants in SDU.

### 3.5. Reasons for Involvement in the SDU

Among the motivations for getting involved in SDU, nearly half of those who used drugs to enhance sexual experiences (46.6%) used them to feel less inhibition and shame (see Table 4). The other frequently reported reasons were to escape or have a good time (42.5%), to take advantage of time and have fun (24.0%), to have sex, perform certain sexual practices, or feel better sexual performance (18.5%), and to help nothing to matter (16.4%). When the results were separated by gender, the reasons most frequently reported by women who were involved in SDU were to feel uninhibited and less shame (50.5%), to get away and have fun (36.6%), and to use their spare time well by having fun (25.8%). In comparison, men reported engaging in SDU mainly to escape and have a good time (52.8%), to disinhibit and experience less shame (39.6%), and to have sex, perform certain sexual practices, or feel that they performed sexually better (24.5%). There were no gender differences in any motivation to practice SDU; only the motivation to escape and have fun approached significance (women = 36.6%, men = 52.8%; χ^2^ = 3.658, *p* = 0.056).

### 3.6. Relationship between Mental Health and Sexualized Drug Use

Finally, we analyzed whether SDU could be associated with symptoms of depression and anxiety. Table 5 presents the comparisons of means by the Student’s *t*-test for the study variables. Those participants who practiced SDU had significantly higher depression scores than those who did not engage in SDU, but this difference is considered small (d = 0.206) (see Table 5). Participants’ anxiety regarding choosing to engage in SDU did not differ significantly. There were no significant differences in levels of depression (women: *M* =14.837, *SD* = 5.479; men: *M* = 12.943, *SD* = 5.709, *t* = 1.973, *p* = 0.054) or levels of anxiety (women: *M* = 12.562, *SD* = 4.788; men: *M* = 11.115, *SD* = 5.055, *t* = 1.710, *p* = 0.089) between the women and men who practiced SDU (data not shown).

## 4. Discussion

Substance use for sexual purposes is a public health problem that affects both MSM and heterosexual men and women. The purpose of this study was to analyze the prevalence and characteristics of SDU in heterosexual women and men. The results show that approximately one in ten heterosexual men and women used drugs for sexual purposes. The data also indicated that the SDU significantly increases the likelihood of reporting unsafe sexual practices and the diagnosis of STIs. Additionally, SDU was related to more reports of depression symptoms. 

This is one of the few studies conducted to date that analyzes heterosexual gender differences among a wide range of SDU correlates (e.g., demographic variables, substances, unsafe sexual practices, STIs, motivations, and mental health outcomes). The results expand on previous research by showing some differences between genders, but the results also show important similarities between genders in terms of overall prevalence, motivations, and mental health outcomes. To date, to our knowledge, it is the first study on SDU among heterosexuals carried out in the Spanish cultural context. A discussion of the implications of these results at a theoretical and applied level follows.

Approximately 12% of the participants reported having used substances for sexual purposes in the past 24 months. This percentage is slightly lower than the results obtained in previous studies carried out in Europe, Australia, and North America with heterosexual samples, which found percentages that ranged between 23% and 31% in men and between 16% and 23% in women [12,62]. This decrease in use could be explained because drug use could be subject to the place where the participants were recruited and the popularity and availability of substances in different cultural contexts and moments [1,23]. In this sense, the prevalence of SDU in MSM also illustrates important oscillations between the different studies, with prevalence rates ranging between 3% and 94% [52]. Although the practice of SDU seems to be limited to a minority of heterosexuals, according to our findings, a non-negligible percentage of heterosexual men and women have used drugs for sexual purposes, as is also the case among MSM [12,63]. Our data indicate that heterosexual adults younger than 36 years of age are more involved than older adults in SDU. This finding is in line with previous studies that have found substance use to be higher among young adults than among older adults [64]. A greater perception of risks [65,66] and greater family and work commitments among older heterosexual adults [67] could partially explain these findings. On the other hand, no significant differences were found based on educational level or income, which suggests that the prevalence of SDU among heterosexuals is similar regardless of socioeconomic status. Around 11% of the participants practicing SDU had not had sex outside the couple relationship (compared to 89% who did not practice it), which would be in line with other studies of the heterosexual population [68,69]. Future interventions should focus on harm reduction in young adults doing SDU, regardless of their gender and sexual orientation.

The findings also reveal that a similar percentage of men (14%) and women (12%) reported having used a drug for sexual purposes. These results are consistent with those obtained in previous prevalence studies, thus indicating a similar prevalence of women and men participating in SDU [11,12,13,70]. Although the proportion of men and women who use drugs to enhance sexual experiences is similar, men who use them seem to use more different types of substances and spend more money to acquire them. Thus, it should be noted that a higher consumption of MDMA, ecstasy, and cocaine takes place among men than among women. This finding is consistent with previous empirical evidence indicating that heterosexual men use cocaine, ecstasy, and MDMA more than heterosexual women do [71,72], which, in turn, could increase men’s exposure to risks associated with STIs, such as unsafe sexual practices. 

Alcohol was the substance that was most frequently consumed among both men and women. These results expand the previous evidence among heterosexuals [64,73]. This use may be due to easy access to alcohol and low perception of risk toward alcohol; however, the social character of SDU would explain the variations in the frequency of consumption of different substances. The rest of the drugs most frequently used for sexual purposes were cannabis, cocaine, ecstasy, and MDMA, which is consistent with the previous findings [13]. 

The results show that the participants who practiced SDU had riskier sexual behaviors than those who did not. Specifically, these risky behaviors included less condom use, greater number of casual sexual partners, and greater use of fetishes or unconventional sexual practices. Our results suggest a positive relationship between substance use and engagement in risky sexual behaviors. These data expand the previous empirical evidence obtained for MSM [34,59,74] by reporting that heterosexual women and men who use drugs for sexual purposes are engaged in higher levels of risky sexual behaviors than those who do not perform an SDU. The present study also examines the differences between SDU, sexual risk behaviors, and gender. In this sense, we find that men more frequently engage in risky sexual practices than do women. This could be attributed to greater reward seeking and less inhibitory control among men [75,76], factors that could contribute to the greater impulsiveness of men to experiment with sexual risk.

Furthermore, our findings show a relationship between SDU and the risk of being diagnosed with syphilis, chlamydia, and others STIs within the last 24 months. These data suggest that people who participate in SDU are diagnosed with an STI more often than people who do not participate. Other studies that focused on drug use and the risk of STIs have shown an increased risk of drug users acquiring STIs, both in men and in women, related to a greater frequency of risky sexual relations [77,78]. For example, in the study by Stevens et al. (2020) [79] of HIV+ MSM, around half of the participants (47%) attributed their seroconversion to drug use. A possible hypothesis may be related to the sensation-seeking personality trait, defined as the need to seek varied and novel experiences and the willingness to take physical and social risks for these experiences [80], leading—as a consequence—to a further diagnosis of STIs.

Regarding the motivations, the most frequently reported were being uninhibited and feeling less shame when engaging in sexual encounters. Similarly, previous evidence indicates that MSM practice SDU to disinhibit, escape, and have fun, among other reasons [34,81]. Men and women reported similar motivations in the consumption of drugs for sexual purposes; however, a more detailed analysis of the types of motivations for participating in the SDU revealed some interesting data. In general, the main motivation for using drugs in a sexual context for men was to escape and have a good time, while women indicated their primary motivation was to be uninhibited and show less shame. These results could be explained by the higher difficulty of women in recognizing and accepting sexual feelings and desires without guilt, fear, or shame due to social pressure [82]. Future studies should continue to study the multicausal and complex reasons why women and men engage in illicit drug use before or during sex.

Finally, the results expand the previous empirical evidence regarding MSM by showing that participation in SDU is associated with more depression, but not with more anxiety. These findings are consistent with several previous studies in MSM that reported a relationship between drug use for sexual purposes and an increased likelihood of presenting with depression outcomes [9,83]. Two hypotheses could be proposed. First, depression could increase the probability of using SDU. In this sense, the SDU could be a maladaptive way of regulating mood. Second, depressive symptoms could be a consequence of the SDU practice. In this sense, SDU could induce feelings typical of depression (e.g., guilt) among those who practice it. Likewise, it should be noted that the association between drug use and depression could be moderated by the type of substances used, frequency of use, and the level of substance dependence. Future studies should analyze the longitudinal relationships between SDU and mental health outcomes, as well as potential moderating variables of this relationship. 

First, these data suggest the importance of developing and strengthening information systems and epidemiological surveillance tools. It is necessary to analyze the SDU phenomenon, its patterns and trends, and its impact on the physical and mental health of the people who practice it, regardless of their sexual orientation. Second, our results suggest the need for group and individual interventions aimed at skills training, risk reduction counseling, and the management of individual cognitions, attitudes, and beliefs related to SDU. Thirdly, affective-sexual programs should be encouraged among risk groups. These programs can improve sexual function by improving people’s attitudes toward their sexuality, encouraging positive acceptance of sexuality in the face of rejection or fear, and teaching alternative interrelationship skills. Finally, these results highlight the need to implement psychological screening programs in essential resources aimed at the early detection of mental health symptoms among those who are involved in SDU in Spain.

This study has several limitations that should be noted. The first is related to the cross-sectional nature of the data; future longitudinal studies should examine the temporal order of the variables. Second, although our sample was large, it was not representative of the Spanish population. Thus, only those adults who used the Internet and were willing to complete an online survey participated in this study; therefore, caution is advised when generalizing the results. In this sense, future studies should replicate and expand the results described here using non-convenience and more representative samples of heterosexual women and men. Third, the evaluation of SDU in the present study included self-reported STI diagnosis. Future studies should have other sources of objective information, such as the medical histories of the participants. Fourth, this study only measured symptoms of depression and anxiety as indicators of mental health outcomes. Future studies should include additional indicators, such as social functioning, addiction, inhibitory control, impulsivity, or measures of personality. Moreover, studies are needed to establish predictive models using techniques such as regression. Due to the high number of analyses calculated in this exploratory study, no adjustments were made to the *p*-value. Once the potentially relevant variables have been identified, future studies should focus on these variables and make the appropriate adjustments. Finally, several limitations of the data collection method should be noted. First, the empirical analyses in this article are based on data obtained through self-reported questionnaires, which may be subject to recall bias, leading to a distortion in the accuracy of recall over time. Likewise, sensitive questions in online surveys make respondents vulnerable to social desirability bias, which could lead to an underestimation of the prevalence of SDU and other variables analyzed in the studies, such as sexual risk behaviors, STI diagnosis, or mental health symptoms.

## 5. Conclusions

This is one of the first studies to analyze SDU among heterosexual women and men and to include indicators of prevalence and individual and health variables associated with SDU. The results suggest that approximately 12% of heterosexual people have been implicated in SDU, and that SDU in heterosexual people is significantly related to risky sexual behaviors, STI diagnosis, and depression symptoms. These data indicate the importance of raising awareness about SDU as a public health problem that is not just limited to MSM. Programs that seek to understand why heterosexuals engage in SDU should be undertaken. It is necessary to design specific identification, education, and prevention programs to reduce the incidence of the most undesirable consequences of SDU in both men and women, regardless of their sexual orientation. Specifically, the intervention should focus on providing information and sharing knowledge for the prevention of STIs and other complications affecting physical and psychological health. Likewise, an approach should be focused on the needs of those who practice SDU, such as the minimization of risk and damages. 

## Figures and Tables

**Table 1:** Associations Between the SDU and the General Characteristics of the Participants.

	SDU		No SDU		χ^2^	p *
	*n*	%	*n*	%		
Gender (*n* = 1177)					1.125	0.289
Women (*n* = 795; 67.5%)	93	11.7	702	88.3		
Men (n = 382; 32.5%)	53	13.9	329	86.1		
Age (years) (*n* = 1177)					12.507	0.006
18–25 (*n* = 928; 78.8%)	117	12.6	811	87.4		
26–35 (*n* = 159; 13.5%)	28	17.6	131	82.4		
36–45 (*n* = 44; 3.7%)	1	2.3	43	97.7		
≥46 (n = 46; 3.9%)	1	2.2	45	97.8		
Country of birth (*n* = 1181)					12.261	<0.001
Spain (*n* = 1137; 96.3%)	134	11.8	1003	88.2		
Other (*n* = 44; 3.7%)	13	29.5	31	70.5		
Education level (*n* = 1155)					0.473	0.492
Below university education (*n* = 626; 54.2%)	83	13.3	543	86.7		
University education (*n* = 529; 45.8%)	63	11.9	466	88.1		
Employment situation (*n* = 1153)					4.621	0.309
Employee (*n* = 319; 27.7%)	45	14.1	274	85.9		
Scholarship holder (*n* = 18; 1.6%)	3	16.7	15	83.3		
Student (*n* = 720; 62.4%)	81	11.2	639	88.8		
Unemployed (*n* = 92; 8.0%)	16	17.4	76	82.6		
Retiree or pensioner (*n* = 4; 0.3%)	0	0.0	4	100		
Economic income (*n* = 838)					0.071	0.790
Less than EUR 1.000 (*n* = 560; 66.8%)	68	12.1	492	87.9		
More than EUR 1.000 (*n* = 278; 32.2%)	32	11.5	246	88.5		
Age of beginning sex (*n* = 1181)					4.905	0.288
<9 (*n* = 11; 0.9%)	2	18.2	9	81.8		
10–14 (*n* = 249; 21.1%)	37	14.9	212	85.1		
15–19 (*n* = 814; 68.9%)	100	12.3	714	87.7		
20–24 (*n* = 47; 4.0%)	2	4.3	45	95.7		
≥25 (*n* = 60; 5.1%)	6	10.0	54	90.0		
Stable or affective partner (*n* =1169)					1.762	0.184
Yes (*n* = 704; 60.2%)	80	11.4	624	88.6		
No (*n* = 465; 39.8%)	65	14.0	400	86.0		
Sex outside the couple (*n =* 1174)					14.514	0.002
Yes (*n* = 82; 6.9%)	21	25.6	61	74.4		
No (*n* = 738; 62.5%)	81	11.0	657	89.0		
No couple (*n* = 354; 30.0%)	44	12.4	310	87.6		

* Fisher’s exact test was used for the variables in which there are less than five cases in the cells.

**Table 2:** SDU Prevalence and Substance Use Among Heterosexual Women and Men.

	Total Participants		Women		Men		*χ* ^2^	*p* *
	*n*	%	*n*	%	*n*	%		
Substances (last 24 months)								
Alcohol	123	10.5	76	9.6	47	12.3	2.076	0.150
Cannabis	84	7.1	49	6.2	35	9.2	3.501	0.061
Cocaine	13	1.1	5	0.6	8	2.1	5.072	0.035
Poppers	8	0.7	4	0.5	4	1.0	1.131	0.283
Ecstasy	17	1.4	7	0.9	10	2.6	5.471	0.019
Viagra^®^/Cialis^®^/Levitra^®^	2	0.2	0	0.0	2	0.5	4.169	0.105
MDMA ^a^	21	1.8	9	1.1	12	3.1	5.944	0.015
GHB/GLB ^b^	1	0.1	1	0.1	0	0.0	0.481	1.000
Methamphetamine	5	0.4	3	0.4	2	0.5	0.130	1.000
Mephedrone	2	0.2	2	0.3	0	0.0	0.963	.822
Heroin	1	0.1	1	0.1	0	0.0	0.481	1.000
Benzodiazepines	2	0.2	2	0.3	0	0.0	0.963	0.822
Other substances	9	0.8	7	0.9	2	0.5	0.433	0.764
Monthly money SDU							15.777	0.002
Any	1031	87.6	702	88.3	329	86.1		
Less than EUR 50	107	9.1	71	8.9	36	9.4		
Between EUR 50 and EUR 100	13	1.1	7	0.9	6	1.6		
Between EUR 100 and EUR 200	5	0.4	0	0.0	5	1.3		
More than EUR 200	5	0.4	1	0.1	4	1.0		
Nothing, they usually invite me	16	1.4	14	1.8	2	0.5		

^a^ 3,4-Methylenedioxymethamphetamine or ecstasy. ^b^ Gamma hydroxybutyrate/gamma butyrolactone. * Fisher’s exact test was used for the variables in which there are less than five cases in the cells.

**Table 3:** Relationship Between SDU and Unsafe Sexual Practices and STIs.

	Total Participants		SDU		No SDU		*χ* ^2^	*p* *
	*n*	%	*n*	%	*n*	%		
Number of sexual partners (last 24 months) (*n* = 1167)							42.667	<0.001
None	360	30.8	30	20.5	330	32.3		
1 to 5	680	58.3	78	53.4	602	59.0		
5 to 10	88	7.5	26	17.8	62	6.1		
10 to 20	29	2.5	8	5.5	21	2.1		
>20	10	0.9	4	2.7	6	0.6		
Condom use frequency (last 24 months) (*n* = 1143)							22.463	<0.001
Never	195	17.1	27	18.5	168	16.9		
Sometimes	165	14.4	33	22.6	132	13.2		
About half the time	97	8.5	17	11.6	80	8.0		
From time to time	87	7.6	16	11.0	71	7.1		
Always	393	34.4	40	27.4	353	35.4		
Not applicable, I have not had penetrative sex with casual partners	206	18.0	13	8.9	193	19.4		
Fetish (*n* = 1129)	160	14.2	37	27.0	123	12.4	21.119	<0.001
BDSM (*n* = 1181) ^a^	94	8.0	21	14.3	73	7.1	9.173	0.002
Fisting (*n* = 1181)	4	0.3	1	0.7	3	0.3	0.580	0.413
Barebacking (*n* = 1181)	64	5.4	16	10.9	48	4.6	9.784	0.002
Serosorting (*n* = 1181)	4	0.3	2	1.4	2	0.2	5.194	0.078
Others (*n* = 1181)	28	2.4	7	4.8	21	2.0	4.147	0.072
STI (last 24 months) (*n* = 1181)	63	5.3	17	11.6	46	4.4	12.905	<0.001
Syphilis	2	0.0	2	1.4	0	0.0	14.092	0.015
Gonorrhea	2	0.2	0	0.0	2	0.2	0.285	1.000
Chlamydia	11	0.9	4	2.7	7	0.7	5.828	0.038
Hepatitis B	1	0.1	1	0.7	0	0.0	7.040	0.124
Hepatitis C	1	0.1	0	0.0	1	0.1	0.142	1.000
HIV ^b^	7	0.6	2	1.4	5	0.5	1.680	0.213
Other STIs	22	1.9	6	4.1	16	1.5	4.521	0.046

*Note*: Data on hepatitis A not presented as no one reported it. ^a^ Bondage and discipline, dominance and submission, and sadism and masochism. ^b^ HIV-1 and HIV-2. * Fisher’s exact test was used for the variables in which there are less than five cases in the cells. Underlined values depict Bonferroni differences between SDU and No SDU.

**Table 4:** Main Motivations for Practicing SDU.

	Total Participants		Women		Men		*χ* ^2^	*p* *
	*n*	%	*n*	%	*n*	%		
1. To evade and/or have fun	62	42.5	34	36.6	28	52.8	3.658	0.056
2. To disinhibit myself and feel less ashamed	68	46.6	47	50.5	21	39.6	1.616	0.204
3. To have sex, perform certain sexual practices, or feel like I’m performing better sexually	27	18.5	14	15.1	13	24.5	2.011	0.156
4. To feel closer to others and be intimate	18	12.3	9	9.7	9	17.0	1.666	0.197
5. To avoid feeling unpleasant feelings, such as sadness, anxiety, or emptiness	13	8.9	7	7.5	6	11.3	0.599	0.548
6. So I don’t feel lonely and isolated	6	4.1	2	2.2	4	7.5	2.495	0.127
7. To isolate myself from the world and that nothing affects me	8	5.5	4	4.3	4	7.5	0.687	0.318
8. It helps me to make sure that everything does not matter to me	24	16.4	14	15.1	10	18.9	0.358	0.550
9. For the feeling that you have to use well the spare time having fun	35	24.0	24	25.8	11	20.8	0.473	0.492
10. None of the above	12	8.2	7	7.5	5	9.4	0.163	0.687

*Note*: * Fisher’s exact test was used for the variables in which there are less than five cases in the cells.

**Table 5:** Relationship Between SDU and Self-Reported Depression and Anxiety.

	SDU			No SDU			*t*	*p*	*d*
	*N*	*M*	*SD*	*N*	*M*	*SD*			
Depression ^a^	146	14.157	5.603	1030	13.082	4.768	2.491	0.019	0.206
Anxiety ^a^	145	12.076	4.919	1028	11.772	4.586	0.739	0.460	0.064

*Note*: ^a^ BSI^®^, anxiety and depression of Brief Symptom Inventory.

## Data Availability

Not applicable.
